# Bioengineered urethral grafts for strictures: a perspective on advancements, functional restoration, and affordability

**DOI:** 10.1097/MS9.0000000000003675

**Published:** 2025-08-01

**Authors:** Shahreena Athar Siddiqui, Maliha Khalid, Muhammad Talha, Aminath Waafira

**Affiliations:** a Dow Medical College, Karachi, Pakistan; b Jinnah Sindh Medical University, Karachi, Pakistan; c King Edward Medical University, Lahore, Pakistan; d The Maldives National University, Malé, Maldives

**Keywords:** 3D bioprinting, hydrogel bio-inks, tissue-engineered grafts, urethral stricture

## Abstract

Urethral stricture is a chronic urological condition that remains challenging to manage due to the limitations of traditional treatments like dilation and urethroplasty, which are prone to recurrence and complications. Tissue-engineered urethral grafts have emerged as a promising alternative, offering durable, functional restoration with reduced donor site morbidity. Innovations in scaffold fabrication, especially 3D bioprinting and hydrogel-based bio-inks, have led to the creation of structurally and biologically compatible constructs that can regenerate native urothelial layers. However, high costs, technical complexity, and limited commercial viability have restricted their widespread clinical use. Future directions should prioritize cost-reduction strategies, scalable biomanufacturing, and head-to-head trials with current treatment standards.

*To the Editor,*

Urethral stricture is a debilitating condition characterized by the narrowing of the urethral lumen, chiefly due to scar tissue formation from trauma, infection, or iatrogenic causes. The luminal constriction, from time to time, may lead to urinary obstruction and other complications, including recurrent infections, bladder dysfunction, and great impacts on quality of life. Effective management is difficult because conventional treatment approaches, such as dilation or urethroplasty, are limited by recurrence and tissue availability^[1]^.

Conventional treatments, such as urethral dilation and Direct Vision Internal Urethrotomy (DVIU), are typically reserved for small (<1.5 cm), simple strictures and provide temporary symptom relief. However, recurrence rates remain high in many cases, for which Open Urethroplasty is a widely used current state of care with its proven efficacy against complex strictures. Despite their established efficacy, these methods are not without drawbacks, including donor site morbidity and potential graft contraction, which can still impose a considerable burden on patients and may not always yield perfect, long-term results^[2]^.

To counter the limitations of traditional interventions, a more promising route for urethral reconstruction has been introduced: Tissue engineering. This approach offers a more long-lasting alternative to conventional grafts, addresses immune rejection and donor site morbidity, and aims to restore original biological function. Longitudinal assessments using cystoscopy and biopsy in human trials have demonstrated that engineered grafts develop normal multilayered urothelium within months^[3]^, correlating with lasting function and durability.

Significant progress in scaffold fabrication techniques, particularly electrospinning and 3D bioprinting, has enabled the creation of constructs with precise structural and mechanical properties. 3D bioprinting technology draws a breakthrough in regenerative medicine, enabling the invention of complex tissue constructs for urethral stricture treatment. This has significantly shifted urethral stricture grafting from substitution to regeneration, enabling the mitigation of recurrence by stimulating the body’s healing mechanisms^[4]^. Further evaluating the domain of urethral grafts, advancements in bio-ink formulations are noticeable, with the emergence of GelMA and dECM-based hydrogels. These are super biocompatible, with their tendency to support the multiplication of urothelial and smooth muscle cells, rudimentary for tissue regeneration and function restoration, nearly simulating the biological extracellular matrix^[4,5]^.

An additional key significant advancement is the strategic use of Extrusion-based bioprinting. This technique has attained predominance due to its capability to manufacture multilayered, tubular scaffolds that closely replicate the structural and mechanical arrangement of the native urethral barricade. These not only have great tensile strength but also long-term physiological function. Other alternate approaches, such as inkjet and laser-assisted bioprinting, offer even better efficacy in reconstructing urethral strictures; however, a significant limitation is posed by their inability to generate large-volume constructs, rendering them non-viable^[4]^.

Despite long-lasting and sustainable outcomes, high production costs and the complexity of tissue-engineered grafting restrict its accessibility among the narrowed-down population groups. This intervention requires cell culture under GMP for weeks, specialized labs, and regulatory approval, and hence, an expensive approach. Moreover, studies have proven tissue-engineered grafts to be non-viable commercially due to an estimated $230 per patient cost advantage only compared to standard grafts^[6]^. This letter to editor is in compliance with the TITAN guideline ^[7]^.

To conclude, bioengineered urethral grafts have been ascertained to be practical in humans for consistently regenerating functional urethras. However, their high costs have prevented this intervention from becoming a routine practice. To promote the development of affordable alternatives, it is recommended that research focus on identifying low-cost scaffolds, accessible cell sources, and point-of-care manufacturing to reduce production time. Moreover, preclinical models should be utilized to directly compare tissue-engineered grafts with current standards in clinical trials.

## Data Availability

No data were generated for this manuscript.

## Research registration unique identifying number (UIN)

Not required.
